# Hospital Notes

**Published:** 1903-08

**Authors:** 


### HOSPITAL NOTES.

The Buffalo General Hospital has added eighteen private rooms and nine semiprivate rooms to its accommodations. These rooms are newly furnished, and completely equipped in every way, and are at the disposal of the physicians of Buffalo for private patients.

The Buffalo Homeopathic Hospital has received from Mrs. William H. Gratwick, a substantial gift in the shape of a new ambulance of the latest construction and fitted with the most modern appliances. It will have rubber tires and ball-bearings axles, and every convenience for the comfort of the patient. A new harness is also included in the outfit.

